# Enhancing the Performance of Perovskite Light-Emitting Diodes via Synergistic Effect of Defect Passivation and Dielectric Screening

**DOI:** 10.1007/s40820-024-01405-5

**Published:** 2024-05-31

**Authors:** Xuanchi Yu, Jia Guo, Yulin Mao, Chengwei Shan, Fengshou Tian, Bingheng Meng, Zhaojin Wang, Tianqi Zhang, Aung Ko Ko Kyaw, Shuming Chen, Xiaowei Sun, Kai Wang, Rui Chen, Guichuan Xing

**Affiliations:** 1 Institute of Applied Physics and Materials Engineering, University of Macau, Avenida da Universidade, Taipa, Macao 999078 People’s Republic of China; 2 Department of Electrical and Electronic Engineering, Southern University of Science and Technology, Shenzhen, 518055 People’s Republic of China

**Keywords:** Synergistic passivation strategy, Defects passivation, Dielectric screening, Perovskite light-emitting diodes

## Abstract

Metal halide perovskites, particularly the quasi-two-dimensional perovskite subclass, have exhibited considerable potential for next-generation electroluminescent materials for lighting and display. Nevertheless, the presence of defects within these perovskites has a substantial influence on the emission efficiency and durability of the devices. In this study, we revealed a synergistic passivation mechanism on perovskite films by using a dual-functional compound of potassium bromide. The dual functional potassium bromide on the one hand can passivate the defects of halide vacancies with bromine anions and, on the other hand, can screen the charged defects at the grain boundaries with potassium cations. This approach effectively reduces the probability of carriers quenching resulting from charged defects capture and consequently enhances the radiative recombination efficiency of perovskite thin films, leading to a significant enhancement of photoluminescence quantum yield to near-unity values (95%). Meanwhile, the potassium bromide treatment promoted the growth of homogeneous and smooth film, facilitating the charge carrier injection in the devices. Consequently, the perovskite light-emitting diodes based on this strategy achieve a maximum external quantum efficiency of ~ 21% and maximum luminance of ~ 60,000 cd m^−2^. This work provides a deeper insight into the passivation mechanism of ionic compound additives in perovskite with the solution method.

**Supplementary Information:**

The online version contains supplementary material available at 10.1007/s40820-024-01405-5.

## Highlights

- Synergistic passivation mechanism of the dual functional compound of potassium bromide for metal halide perovskite films, that is through chemical bonding and physical screening towards the various types of defects.
- Metal-ion additives can simultaneously passivate the defects of halide vacancies with bromine anions and dangling bond defects with metal cations.
- Achieve the maximum external quantum efficiency of ~21% and maximum luminance of ~60,000 cd m^−2^.

**Supplementary Information:**

The online version contains supplementary material available at 10.1007/s40820-024-01405-5.

## Graphical abstract

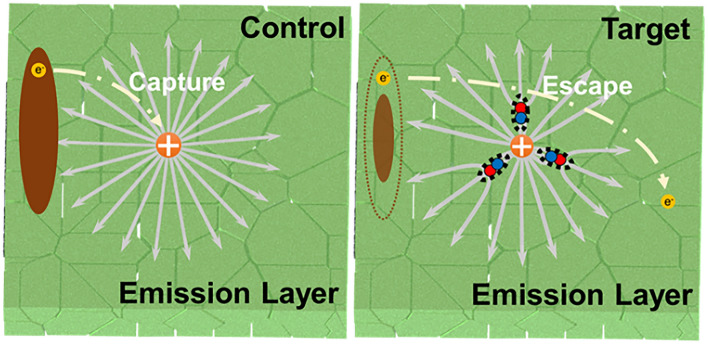

## Introduction

Metal halide perovskites, with their exceptional optical properties such as tunable emission, high color purity, and high photoluminescence quantum yield (PLQY), have emerged as compelling contenders for next-generation light-emitting diodes [1–8]. Within the class of perovskites, there is quasi-two-dimensional (quasi‐2D) Ruddlesden-Popper (RP) perovskite [9–12], characterized by a quantum well structure represented by the general formula (RNH_3_)_2_A_*n*−1_B_*n*_X_3*n*+1_ (where *n* = 1 corresponds to a pure two-dimensional layered structure, *n* = ∞ represents a three-dimensional structure, and *n* = a specific integer denotes a quasi‐2D layered structure). By incorporating cations that do not fit into the cuboctahedra cavities between BX_6_ octahedra, the cubic symmetry is disrupted, resulting in the division of the original 3D inorganic lead halide layer into slices oriented along < 001 > or < 110 > directions [8]. This increased structural flexibility in low-dimensional materials provides new possibilities for constructing desired structures and further optimizing the optoelectronic properties of perovskite materials. For example, it enables the construction of efficient energy funneling channels in the perovskite film, which significantly improves the efficiency of radiative recombination and boosts the progress of the performance of perovskite light-emitting diodes (PeLEDs). Additionally, the higher formation energy of low-dimensional perovskites and the hydrophobic nature of organic ligands allow them to have high moisture stability [13, 14].

Defects are considered to be the most critical factors that hinder device performance and operational stability, and a great amount of effort has been devoted to understanding their characteristics and suppressing their formation [15–17]. In general, the defects in metal halide perovskite (ABX_3_) include 12 native point defects: the vacancies of *V*_*A*_, *V*_*B*_, and *V*_*X*_; the interstitials of *A*_*i*_, *B*_*i*_, and *X*_*i*_; and anti-site occupations of *A*_*B*_, *A*_*X*_, *B*_*A*_, *B*_*X*_, *X*_*A*_, and *X*_*B*_ [18]. These defects can be classified into two categories: (1) deep-level defects, that is, the defects associated with divalent metal cations, such as *B*_*i*_, *B*_*A*_, *B*_*X*_, and *X*_*B*_, which have high formation energies, (2) shallow-level defects, that is, the other defects mentioned before, which have low formation energies. As a result, shallow defects, such as halide vacancies (*V*_*X*_), lead vacancies (*V*_*B*_), and formamidine vacancies (*V*_*A*_), are considered to be the major defects that are detrimental to the efficient radiative recombination [19]. Various defect passivation methods, including anti-solvent technique, additive engineering, composition engineering, film post-treatment, etc., have been extensively studied [20–24]. The addition of alkali metal cations to perovskite precursors has been confirmed as an effective approach to improve the optoelectronic properties and moisture stability of perovskite films [25–32]. Yang et al. demonstrated that the introduction of potassium ions can occupy specific sites on the grain boundary of perovskite, which can suppress the non-radiative recombination and enhance charge carrier transport in perovskite nanocrystals [33]. Zhou's group found that the addition of K^+^ could suppress the defect states by modulating the crystallization dynamics, and limiting the nucleation of high-*n* phases while promoting the growth of low-*n* phases, resulting in a more uniform phase distribution and enhanced energy transfer efficiency [34]. In addition, considering the ionic properties of perovskite crystals and the electro-positivity of the alkali metal ions, the Coulomb interactions between them on the radiative recombination efficiency of perovskite films should not be overlooked.

In conventional semiconductors, Coulomb interactions between carriers and charged defects play a crucial role in carrier diffusion and transport [18]. The Coulombic interactions, whether characterized by attraction or repulsion, are contingent upon the charged properties inherent in the defects. Exerting control over the Coulomb interaction between carriers and defects through the dielectric screening effect holds immense significance in mitigating the deleterious impact of defects in intrinsic perovskites [35]. The dielectric screening mentioned here refers to the fundamental changes of the Coulomb interactions and substantial reduction of defect capture cross section, enabled by regulating the dielectric properties of perovskite films. However, the dielectric screening effect on defect passivation by perovskite composition engineering has not yet been explored to optimize the optical properties and performance of light-emitting devices.

Here, we demonstrate a synergistic passivation strategy using a dual-functional compound of potassium bromide to improve the morphological and optoelectronic properties of perovskite films, taking advantage of the compositional and electrical properties of this ionic compound. Through introducing the potassium bromide into the perovskite films, on the one hand, the defect of bromine vacancies and lead dangling bonds was passivated with the extrinsic bromide. On the other hand, the incorporation of charged potassium ions induces changes in the localized electric field distribution, effectively screening charged defects on the perovskite surface and suppressing carrier trapping. The combined operation of these mechanisms significantly improves the PLQY of perovskite thin films from 66% to an impressive 95%. This synergistic strategy reduces the density of defect states and reduces the possibility of carrier trapping by charged defects, thereby effectively inhibiting non-radiative recombination. As a result of the optimization, the performance of the device has been significantly improved, with a maximum external quantum efficiency (EQE) of approximately 21%, a maximum current efficiency of 90.98 cd A^−1^, and a maximum luminance of up to 60,000 cd m^−2^. The PeLEDs have excellent repeatability and a stable emission peak at 532 nm.

## Experimental Section

### Materials

We obtained poly(N-vinyl carbazole) (PVK) and polyvinyl pyrrolidone (PVP) from Lumtec for our research. Additionally, Xi'an Yuri Solar Corp supplied us with FABr, PEABr, PbBr_2_, and MACl. TPBi and LiF were purchased from Guangdong Aglaia Optoelectronic Materials Corp. As for dimethyl sulfoxide (DMSO) and ethyl acetate, we procured them from Acros. It is important to note that all the chemical materials were utilized without any further purification.

### Preparations of Quasi-2D Perovskite Layers

All quasi-2D perovskite layers are acquired through the process of spin-coating. The precursor solution is meticulously crafted by amalgamating PbBr_2_, FABr, PEABr, and MACl in precise proportions of 5:4:2:0.5, within the solvent DMSO. Notably, MACl serves as an additive, imparting its prowess in enhancing the crystallization process. To achieve bulk passivation, a solution of 0.1 M KBr can be directly infused into the perovskite precursor solution, harmoniously blending with other precursor components. Subsequently, the solution with a concentration of 0.2 M (in terms of Pb^2+^) is spin-coated at a rapid velocity of 6000 rpm for a duration of 25 s. During the spin-casting procedure, a measured quantity of 100 µL of ethyl acetate is delicately poured onto the film. Finally, the film undergoes annealing on a hot plate, meticulously set at a temperature of 90 °C for 60 min.

### Device Fabrication

The glass substrates coated with indium tin oxide (ITO) undergo a meticulous cleaning process involving sequential treatment with detergent, distilled water, acetone, and ethyl alcohol using an ultrasonic cleaner. Subsequently, the pre-cleaned substrates are subjected to a 5 min plasma treatment to render the surface hydrophilic. Following this, the substrates are carefully transferred into a glove box filled with nitrogen. A solution of PVK (10 mg mL^−1^ in chlorobenzene) is then spin-coated at a speed of 4000 rpm for a duration of 40 s, and the resulting films are baked at 120 °C for 30 min. Following this step, a solution of PVP (1 mg mL^−1^ in isopropyl alcohol) is spin-coated at the same speed and duration, with the films subsequently baked at 120 °C for 10 min. The perovskite film is then deposited onto the PVP layer. Finally, TPBi (55 nm), LiF (1 nm), and Al (100 nm) are sequentially deposited through thermal evaporation. The active area of the device measures 0.04 cm^2^.

### Characterizations

UV–visible absorption spectra were acquired utilizing the Perkin Elmer 950 spectrophotometer. The PLQY measurements were conducted employing the Hamamatsu Quantaurus-QY apparatus, model no. C11347. Steady-state and time-resolved photoluminescence analyses of the perovskite were performed using the PicoQuant FluoTime 300, equipped with a picosecond pulse laser (360 nm, LDH-P-C-360) featuring a pulse width of 40 ps. Time-resolved photoluminescence measurements were carried out employing a time-corrected single photon counting (TCSPC, PicoHarp 300E) module, incorporating a photomultiplier (PMA-C 192-M) detector. X-ray diffraction analysis was conducted utilizing a Rigaku SmartLab X-ray diffractometer with Cu κα radiation as the source. *C*–*V* measurements were recorded employing a Keithley 4200A parameter analyzer at 1 kHz with an AC amplitude of 50 mV. Capacitance-frequency (*C-f)* measurements were performed utilizing a potentiostat (PGSTAT302N, Autolab) equipped with a frequency analyzer module, encompassing a frequency range from 10 to 10 MHz, and an AC amplitude of 100 mV under dark conditions. The absolute dielectric permittivity was ascertained to be 8.85 × 10^−12^ F m^−1^, and the device area measured 0.04 cm^2^. *J-V-L* curves were obtained utilizing an Ocean Optics system, which included a Keithley 2400 source meter, an integrating sphere (FOIS-1), and a QE65 Pro spectrometer. Before employment, the integrating sphere underwent calibration using a radiometric calibration light source (HL-3plus). The morphology of the perovskite films and the cross-sectional SEM characterization were scrutinized utilizing the ZEISS Gemini 300 instrument. AFM images were obtained employing the MFP-3D Stand Alone atomic force microscope. X-ray photoelectron spectroscopy (XPS) measurements were performed using the Thermo Fisher ESCALAB Xi + expanding X-ray Photoelectron Spectrometer. Transient absorption (TA) spectroscopy was conducted utilizing the Ultrafast System HELIOS TA spectrometer, wherein the pump wavelength of 360 nm was generated via a Light Conversion TOPAS-C optical parametric amplifier. The corresponding laser source was derived from a Coherent Legend regenerative amplifier (150 fs, 1 kHz, 800 nm), seeded by a Coherent Vitesse oscillator (100 fs, 80 MHz). Broadband probe pulses (420–780 nm) were generated by focusing a minute portion (approximately 10 μJ) of the primary 800 nm laser pulses into a 2 mm sapphire plate.

## Results and Discussion

### Optical Properties

**Fig. 1:**
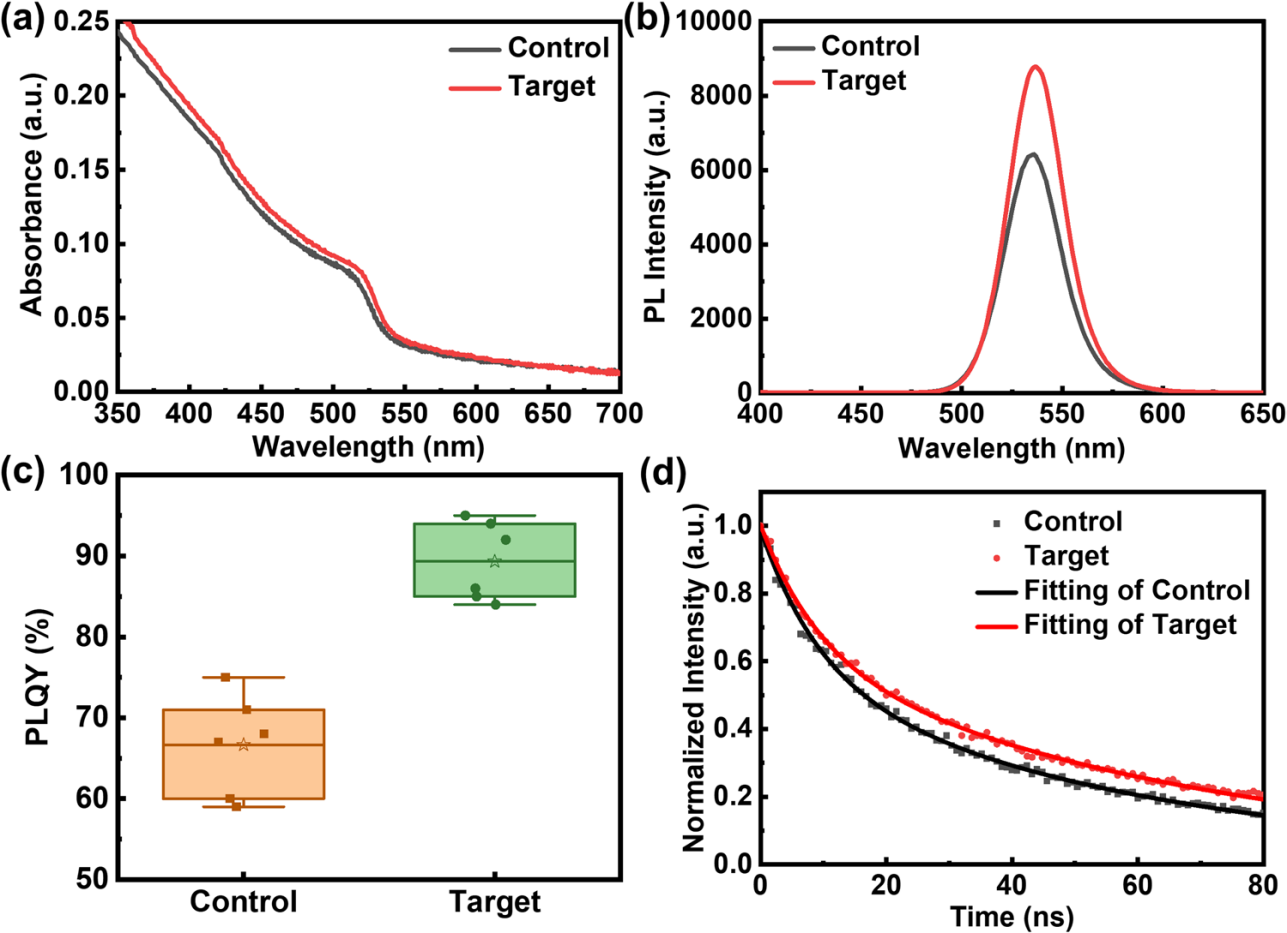
Optical properties of the perovskite films. **a** UV–vis absorption spectra. **b** Steady-state PL spectra. **c** PLQY distribution of the control and target perovskite thin films, measured under illumination with a 360 nm laser. **d** TRPL decays of the control and target samples, excited by a 360 nm laser

The control perovskite films of PEA_2_(FAPbBr_3_)_4_PbBr_4_ were prepared by spin-coating method with a stoichiometric mixture of PEABr, FABr, and PbBr_2_ dissolved in DMSO with a molar ratio of 2:4:5. The target perovskite films were prepared by introducing a specific amount of potassium bromide (KBr) additives into the precursor (see details in the experimental section). To investigate the effect of KBr on the optical characteristics of the film, UV–visible absorption spectroscopy, and photoluminescence (PL) spectroscopy were performed. Figure 1a illustrates that the pristine perovskite film shows a distinct absorption peak at 513 nm (*n* ≥ 5). In comparison, the introduction of an appropriate amount of KBr results in a slight increase in absorption, accompanied by a redshift of emission to 518 nm. The enhanced absorption could be ascribed to the increased film thickness which will be verified in detail later. The redshift of band edge absorption may be attributed to the slightly larger size of three-dimensional-phase (*n* = ∞) crystals with weaker quantum confinement. The increase in crystal size can be attributed to the passivation of dangling bonds by potassium ions, as well as their stronger ionic bonding energy partially replacing the long-chain ions of PEA^+^. Interestingly, during the spin-coating process, it is found that the target perovskite requires a delayed dropwise of the anti-solvent due to the prolonged crystallization process with the existence of KBr. Therefore, the slow crystallization process provides the necessary conditions for grain growth, leading to the larger size of three-dimensional perovskite with KBr as mentioned above. Meanwhile, the crystal quality was improved. Figure 1b shows a comparison of the PL spectra of the control and target perovskite thin films. A significant increase in PL intensity after the addition of KBr accompanied by a slight redshift can be observed, which is consistent with the observation in the absorption spectra. The increase in PL intensity indicates preferable radiative recombination of charge carriers in the target perovskite film. To eliminate the influence of the films’ thickness, we conducted PL experiments on control and target thin films at different thicknesses. As shown in Fig. S1, the PL intensity slightly increased with the film thickness from approximately 25–50 nm. However, the impact arising from thickness variations is considerably smaller than the influence induced by KBr doping, thereby confirming that the doping of KBr indeed enhances the PL intensity and substantiating the deduction illustrated in Fig. 1b. To further confirm the improvement of emission efficiency, PLQY measurement on the samples was conducted (Fig. 1c). The target perovskite thin film exhibits a significantly improved PLQY, achieving a maximum value of 95%. The improvement of PLQY from an average of approximately 66.5% in the control perovskite film to approximately 89.5% after treatment suggests a noticeable reduction of non-radiative recombination. Furthermore, we performed time-resolved photoluminescence (TRPL) measurements to study the PL lifetime of the perovskite thin film (Fig. 1d). After KBr doping, the average lifetime of the film was significantly extended from 53.7 to 63.1 ns (Table S1). This result can be attributed to the suppression of defects and non-radiative pathways. In order to gain a deeper understanding of the physical mechanisms behind the performance enhancement of perovskite films, transient absorption (TA) characterization was conducted to investigate the phase composition within the perovskite film. As depicted in Fig. S2a, b, only one ground-state bleach (GSB) peak is detected in both the control and target films (excited at 360 nm), which is attributed to the *n* ≥ 5 phase. This results that the perovskite films exhibit a predominantly uniform phase composition with high-*n* values. Interestingly, the GSB peak in the target film exhibited a slight redshift compared to the control film (Fig. S2c, d), in agreement with the steady-state absorption results.

### Defect Passivation

**Fig. 2:**
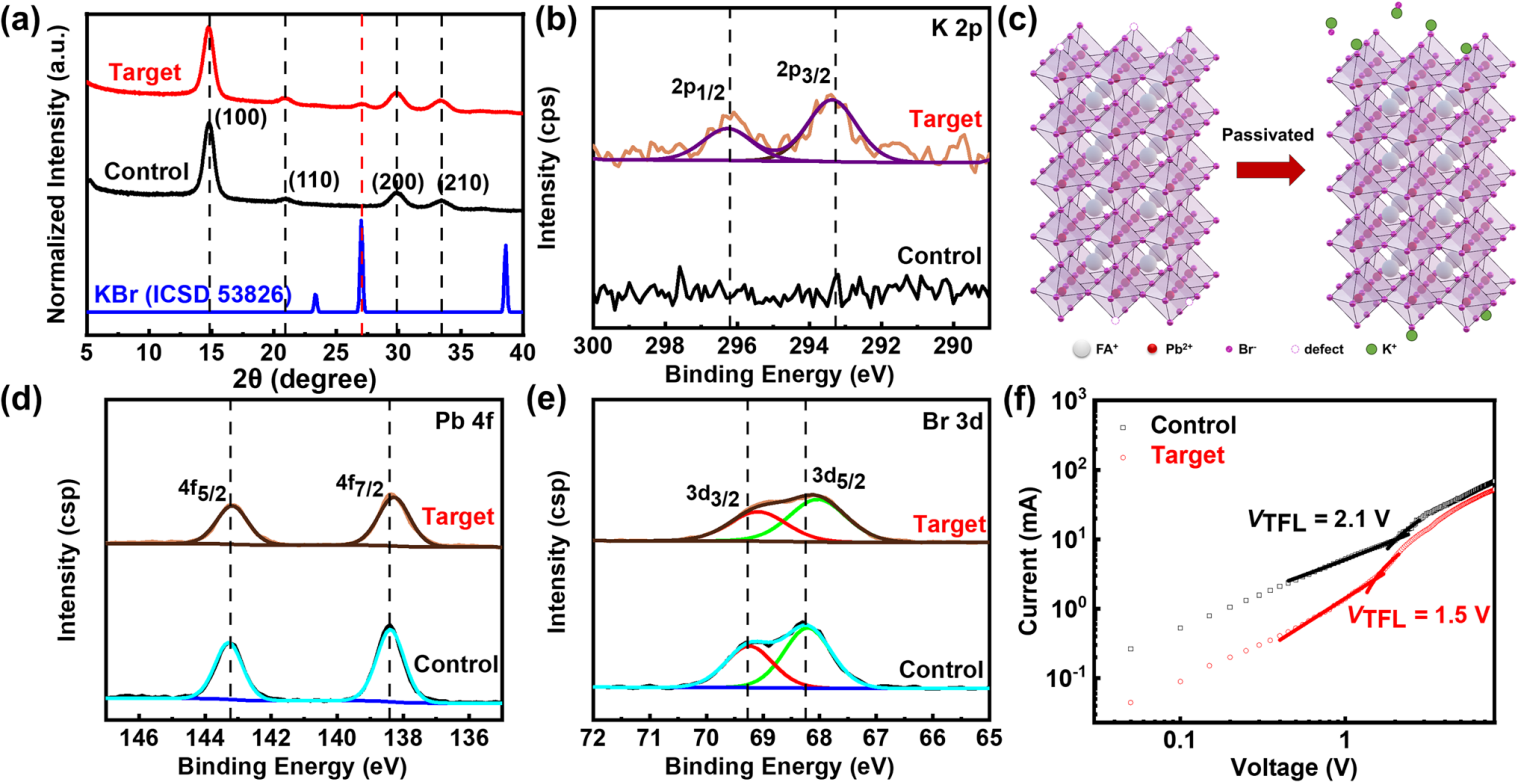
**a** XRD patterns of perovskite thin films.** b** XPS spectra of K 2*p* signals of the control and target perovskite films. **c** Schematic illustration of KBr passivation. XPS spectra: **d** Pb 4*f* signals and** e** Br 3*d* signals of control and target perovskite films. **f** Space charge limited current (SCLC) measurements

In order to investigate the influence of potassium ions on the perovskite crystal structure, the X-ray diffraction (XRD) measurement was then performed. The XRD patterns of control and target perovskite films deposited on quartz substrate are illustrated in Fig. 2a. There are four obvious diffraction peaks at 14.72°, 20.88°, 29.76°, and 33.40°, representing the reflection signals from the crystal plane of (100), (110), (200), and (210), respectively. Additionally, it should be noted that a new peak at 27° appears in the target perovskite film, which is related to the diffraction peak of pure KBr crystals (shown in Fig. 2a blue line), demonstrating the formation of aggregated KBr in the perovskite thin film. Based on previous research findings, it has been established that potassium ions (K^+^) do not replace the A-site ions within the three-dimensional lattice of perovskite due to their diminutive ionic radius [36]. No additional peaks were observed in the XRD patterns (Fig. 2a), confirming the derivative associated with KPbBr_3_ was not formed, which is consistent with the observation in the previous report. According to the density functional theory (DFT) simulation in the previous report, if the minute quantities are incorporated into the perovskite lattice, they tend to occupy the interstitial sites, that is, referring to the positions between the two A-site points, which would lead to lattice expansion [37]. Therefore, to confirm whether the introduced K^+^ gets into the interstice of the lattice and becomes a potential defect, we further examined the structure characteristics of perovskite films with varying concentrations of KBr. As shown in Fig. S3, with the increase in doping concentration, the intensity of the peak at 27° gradually amplifies, suggesting the precipitation of surplus KBr. However, the positions of the primary peaks of perovskite with various K^+^ amounts are almost identical to those of the pristine sample, indicating that doping does not induce lattice expansion or interstitial occupation in this case. This result further confirms that K^+^ ions do not replace the FA^+^ ions at the A-site or enter into lattice spacing, but are located at the grain boundaries. Furthermore, the intensity of XRD diffraction peaks of regulated samples is slightly enhanced, indicating the moderate doping of KBr can improve the crystallinity of perovskite films.

X-ray photoelectron spectroscopy (XPS) measurements on the potassium element confirmed the presence of K^+^ ions in the target perovskite film, as compared to the control film (Fig. 2b). The perovskite film on the grain boundaries contains abundant bromide anions and potassium cations, which can effectively passivate surface defects, as depicted in Fig. 2c. Typically, perovskite defects are mainly concentrated on the surface, including halide vacancies and halide dangling bonds [38]. When KBr is added to the perovskite film, the excess bromide anions can occupy the halide vacancies, while the potassium cations have the potential to interact with the halide dangling bonds, thus passivating the defects.

In order to identify the interactions between perovskite and potassium derivative, the XPS of Pb 4*f* and Br 3*d* is compared in Fig. 2d, e. The characteristic peak of Pb 4*f*_5/2_ and Pb 4*f*_7/2_ of the control perovskite film posited at 143.2 and 138.3 eV, respectively, and these peaks show a slight shift toward lower binding energy after KBr doping (Fig. 2d), indicating that the chemical environment was changed after the introduction of K^+^. The shift in the Pb 4*f* signals suggests that the electron cloud density around Pb^2+^ is increased after moderate KBr doping, which can be attributed to the contribution of the bonding of extrinsic Br^−^ with Pb^2+^. Similarly, the signals of the Br 3*d*_3/2_ and Br 3*d*_5/2_ of target perovskite films also shift toward lower binding energy compared with that of control film (Fig. 2e), indicating an increase in electron cloud density on account of electrostatic attraction between K^+^ and Br^−^. Additionally, excessive KBr doping leads to the aggregate precipitation of potassium bromide due to the stronger binding energy between potassium ions and bromine (Fig. S4, Table S2). In Fig. S4, with the increase in concentration of KBr, the peaks of Br 3*d* undergo an initial shift toward lower binding energy, followed by a subsequent shift toward higher binding energy. This phenomenon can be attributed to the fact that the uniform dispersion of potassium bromide is conducive to the interaction between potassium bromide and perovskite when KBr is present in small quantities. However, when the volume of KBr is relatively large, it tends to aggregate, leading to a weakened passivation effect on the perovskite. Consequently, the chemical environment of Br in this case is similar to the control sample. The evolution of the Pb 4*f* peak position exhibits a similar trend with the variation of Br 3*d* peaks, indicating the excess (≥ 0.15 M) potassium bromide has little passivation effect on perovskite. The peak intensity of K 2*p* gradually increases with the increase in KBr concentration, verifying an increasing content of potassium element in the thin film. This observation further confirms the crystallization and aggregation of excess potassium ions in the perovskite films with a large amount of doping.

In summarizing the preceding conclusions, we can infer that the doping of KBr can effectively passivate the surface defects (e.g., bromine vacancies, bromine dangling bonds) at the grain boundaries rather than enter into the lattice. In order to quantitatively assess the passivation of defects in perovskite films by KBr, Space Charge Limited Current (SCLC) measurements were conducted (Fig. 2f) to determine the defect state density. The current–voltage (*J*–*V*) characteristics of the devices were measured in the absence of light, employing the device structure of Indium Tin Oxide (ITO)/perovskite/MoO_3_/Ag. The defect state density of the perovskite film can be calculated using the equation:

1 $$N_{t} = \frac{{2\varepsilon_{0} \varepsilon_{r} v_{{{\text{TFL}}}} }}{{qL^{2} }}$$

where *V*_TFL_ represents the threshold voltage for defect filling, *L* is the film thickness, *q* denotes the electron charge, and *ε*_*r*_ and *ε*_*0*_ refer to the dielectric constant and vacuum dielectric constant, respectively. A lower *V*_TFL_ generally indicates a reduced defect density in the film. To determine the trap state density, *ε*_*r*_ is calculated utilizing *ε*_*r*_ = (*CL*)/(*ε*_*0*_*S*), where the geometric capacitance *C* can be obtained from the capacitance-frequency (*C-F*) curve at frequencies around 10^3^–10^5^ Hz, which will be explained in detail later. The calculation reveals that the defect state density decreases from the initial 2.82 × 10^17^ to 1.39 × 10^17^ cm^−3^, which is reduced by half after the passivation.

It is found that such a small reduction in defect state density is not sufficient to support the large increase in PLQY. In order to get an in-depth analysis of changes in recombination behaviors, we estimated the radiative and non-radiative recombination constant (*k*_*r*_*, k*_*nr*_) of the perovskite films using the equation of PLQY = *k*_*r*_/ (*k*_*r*_ + *k*_*nr*_) and *τ*_avg_ = (*k*_*r*_ + *k*_*nr*_)^−1^ (Table S3). The *k*_*nr*_ is obviously decreased from 0.624 to 0.166 (× 10^7^ s^−1^) after passivation. Since the pump fluence used in PLQY and TRPL measurement is relatively low, the Auger recombination is negligible in this case. Therefore, the non-radiative recombination should be dominated by the charge carrier trapping at defects. Nevertheless, the calculated decrease in non-radiative recombination rate (- (*k*_*nr* (Target)_—*k*_*nr* (Control)_)/ *k*_*nr,* (Control)_) is much larger than the decrease in defect density (- (*N*_*t* (Target)_—*N*_*t* (Control)_)/*N*_*t* (Control)_). In other words, based on the above-mentioned approximation and defect density decrement result, the calculated PLQY of the target film is about 80%. However, the measured PLQY of the target sample averaged as high as 89.5%, exceeding the calculated value. This indicates that, in addition to defect passivation, there are other reasons within the system that contribute to the reduction of non-radiative recombination.

### Dielectric Screening

**Fig. 3:**
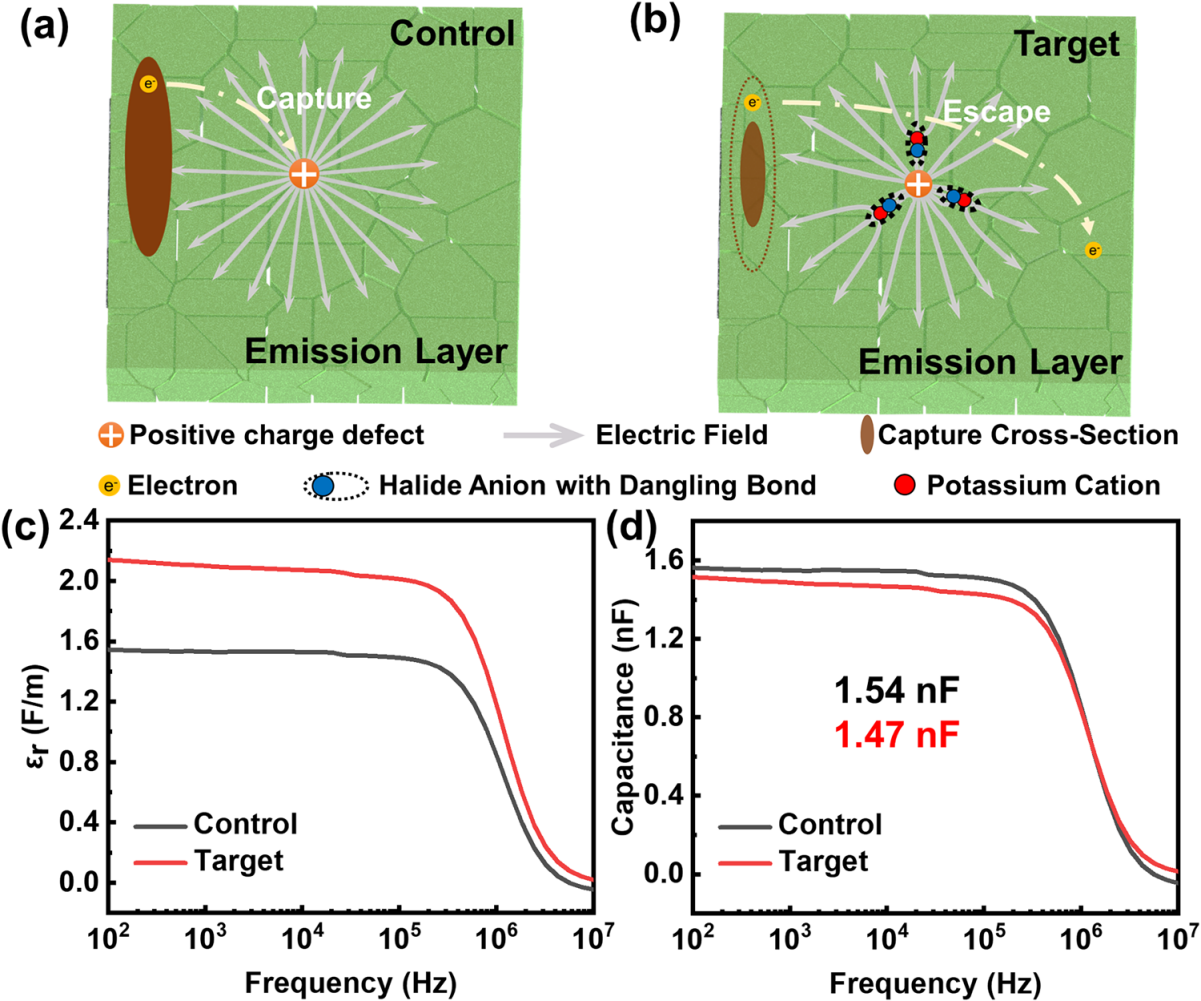
Schematic diagram of the defect capture process in **a** control and **b** target perovskite films. For simplicity, we only show the process related to electrons, but it's important to note that the same principle applies to holes as well. **c** Dielectric constant for control and target perovskite films as a function of frequency. **d** Capacitance–frequency (*C–f*) curve of control and target perovskite devices

It is believed that a significant amount of non-radiative losses is due to the presence of charged trapping states within the perovskite layer, which are typically associated with charged ion defects [29]. As mentioned before, the introduction of KBr provides abundant bromide ions to fill bromine vacancies, combining with the strong binding energy between potassium ions and bromine, enabling the passivation of dangling bonds. We have previously discovered that defect passivation is not the only factor leading to the decrease in non-radiative losses. Considering the electro-positivity of potassium ions, it can be reasonably assumed that the potassium ions can alleviate the impact of the charged defects at the grain boundary via dielectric screening. As Fig. 3b demonstrates, potassium ions form electrically neutral complexes after passivating dangling bonds. When the defect center carries a negative charge, the positively charged potassium ions are naturally attracted to it. Conversely, if the defect center is positively charged, there is a reversal, resulting in the negatively charged side closing the center. Furthermore, previous studies have demonstrated that the variations in the dielectric response of films are almost independent of halide ions [35]. It can thus be deduced that potassium ions have a significant impact on the spatial distribution and response mode of space charge within the perovskite material, consequently affecting the dielectric constant of the perovskite. As a result, it is hypothesized that the alteration in the dielectric constant leads to changes in the localized electric fields, causing dielectric screening. This screening effect reduces the influence of charged defects, rendering them ineffective in affecting the charge carriers. Consequently, it enhances charge carrier transport within the films (Fig. 3a, b). In order to demonstrate the variation of localized electric fields, we conducted the measurement of dielectric response in the frequency range below 10^6^ Hz, as depicted in Fig. 3c. To illustrate this phenomenon, the typical process of electron capture should be reconsidered. When electrons encounter defects with positive charges, they can be captured by these positively charged defects through the Coulomb attraction. In the case of Coulomb attractive defects, the required Coulomb potential energy is equal to the thermal energy of the electrons. By considering this relationship, we can determine the electron capture cross section *σ*_*-*_ as a reference [35], given by

2 $$\sigma_{ - } = \frac{{q^{4} }}{{16\pi \left( {\varepsilon_{r} \varepsilon_{0} k_{B} T} \right)^{2} }}$$

where *q* represents the electron charge, *ε*_*r,*_ and *ε*_*0*_ are the dielectric constants and vacuum dielectric constant, *k*_*B*_ denotes the Boltzmann constant, and *T* signifies the temperature. From this equation, it is apparent that *σ*_*-*_ will decrease with the increase in the dielectric constant at a constant temperature; in other words, an augmented dielectric constant of the perovskite material would lead to a reduction in the defect capture cross section. This variation can be understood as a dielectric screening effect, which weakens the defect capture behavior of charge carriers, rendering them "invisible" to the charge carriers, or in other words, the defect invisible. This reduced trapping cross section will enhance charge carrier transport within the perovskite film. The dielectric constant, which is the ratio of the material's dielectric constant to the vacuum dielectric constant, describes the material's ability to accommodate electric flux. It is also frequency-dependent, meaning it varies with the applied frequency. Therefore, to ascertain the dielectric constant *ε*_*r*_, we first measure the capacitance *C* as a function of frequency, as exquisitely demonstrated in Fig. 3d. Subsequently, we conducted a multipoint measurement to obtain the average film thickness of control and target samples, which were found to be 35 and 50 nm, respectively, as illustrated in Fig. S5. Finally, we calculate *ε*_*r*_ based on the equation *ε*_*r*_ = (*CL*)/(*ε*_*0*_*S*), where *C* is the capacitance and *S* is the area. *ε*_*r* (Control)_/*ε*_*r* (Target)_ = 80.08/94.08, indicating an increase in the dielectric constant of perovskite films after doping of KBr. Combined with the previous equation, this suggests a reduction of the defect capture cross section, enabling effective screening of charged defects and thus improving charge carrier transport.

### Devices Performance

**Fig. 4:**
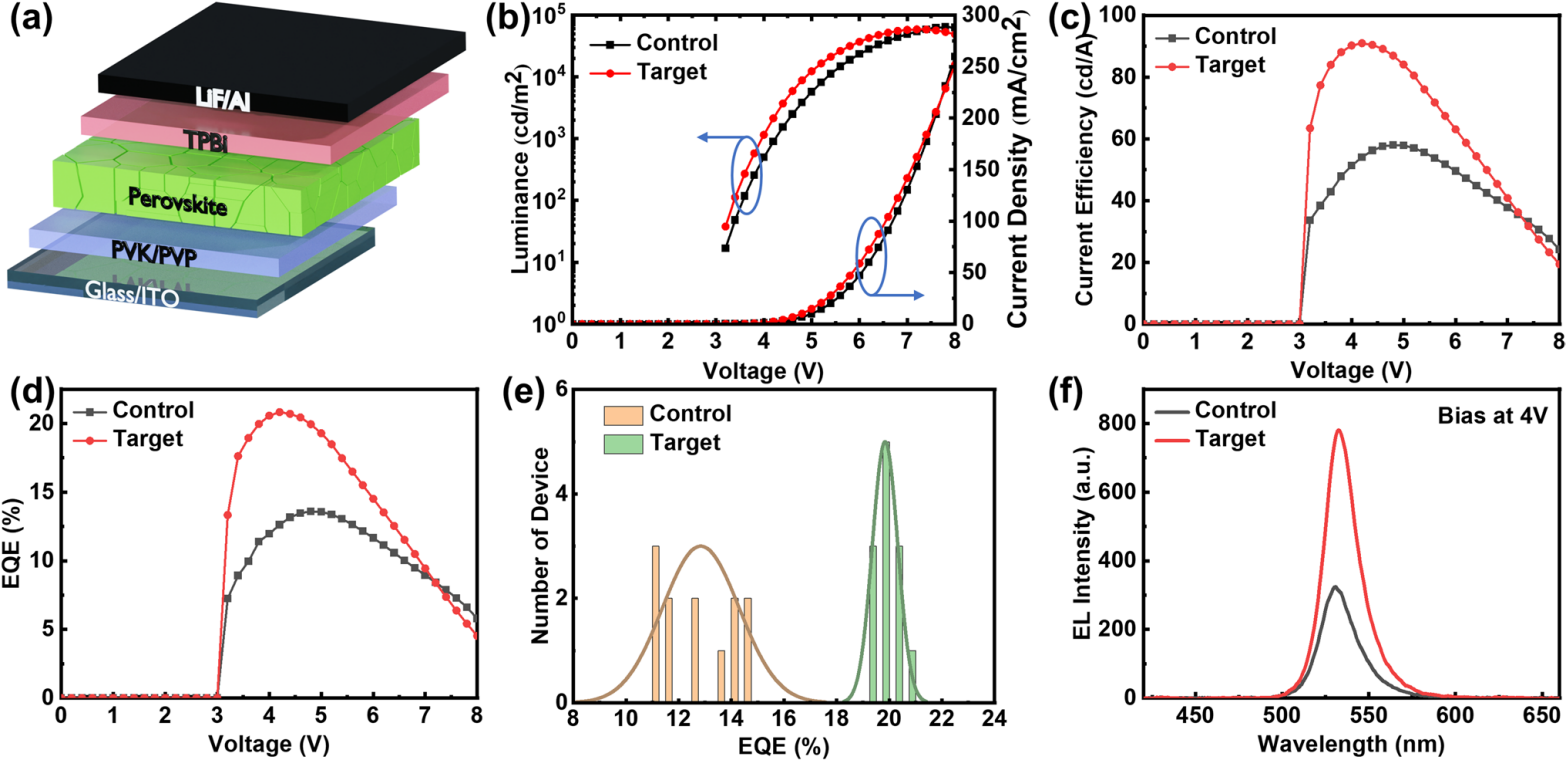
**a** Device structure of the PeLEDs. **b** Current density–luminance–voltage (*J–L–V*), **c** current efficiency (CE–*V*), and **d** efficiency –voltage (EQE–*V*) curves for control and target PeLED devices. **e** Histograms of 12 devices for each control and target PeLEDs. The average EQE of control and target PeLEDs is 12.84% and 19.84% with a relative standard deviation of 11.3% and 2.43%, respectively. **f** EL spectra of control and target PeLEDs

Based on the above understanding, we then fabricated PeLEDs based on the potassium bromide-modified perovskite films and compared their performance with that of the pristine perovskite-based devices. The device structure of the PeLEDs consists of ITO/PVK/PVP/perovskite/2,2′,2″-(1,3,5-benzinetriyl) tris(1-phenyl-1H-benzimidazole) (TPBi)/LiF/Al, where the perovskite serves as the emissive layer, and PVK and TPBi act as the hole transport layer (HTL) and electron transport layer (ETL), respectively. The complete device structure is depicted in Fig. 4a, while the SEM cross-sectional images (Fig. S6) showcase the layered structure of the fabricated PeLED devices. As shown in Fig. S6a, b, it is revealed that the thickness of the perovskite emissive layer after doping has a slight increase, which is consistent with the results presented in Fig. S5a. Figure 4b is the current density and luminance of devices as a function of applied voltage. The KBr-modified perovskite exhibits a higher electroluminescence intensity at a specific applied voltage, meanwhile, it enables a faster attainment of maximum brightness. The current density of the target PeLED is slightly higher than the pristine one, which could be attributed to the smaller contact resistance in the modified PeLED. In order to estimate the interface contact between perovskite films and the ETL layer, the surface morphology is characterized with SEM (Fig. S7) and AFM (Fig. S8). From Fig. S7a, it is evident that the control perovskite film exhibits noticeable cracks on the surface, along with some bright white strips. These white strips may correspond to low-dimensional perovskite phases. In Fig. S7b, the target film shows less conspicuous cracks, indicating a smoother surface compared to the control film. The white particles observed in target film can be attributed to the aggregation of KBr, which is consistent with that revealed in the XRD results. To further validate the positive effect of KBr on the surface morphology of the perovskite film, we conducted AFM characterization. Figure S8 shows that the surface height scale of the thin film decreased from 0–110 nm in the control perovskite film to 0–22 nm in the target, with a reduction of the average surface roughness from 1.7 to 0.27 nm. These results indicate that the surface morphology of the target perovskite films is smoother and more uniform compared to the control films. The enhanced film quality is attributed to slowing down crystallization rate after the incorporation of K^+^, due to the competition between positive charged K^+^ and FA^+^, as well as PEA^+^ in the precursor film, which originated from the Coulomb interaction between K^+^ and PbBr_6_^4–^ frames [34]. The slow crystallization process allows the uniform growth of perovskite nanocrystals, and facilitates the formation of homogeneous and smooth film. The homogeneous and smooth nature of the films facilitates the transport of charge carriers and enhances carrier mobility within the perovskite film. Consequently, this leads to a slightly larger current density under the same voltage. Consequently, the maximum current efficiency (CE) of the PeLED increased from 58.00 to 90.98 cd A^−1^ (Fig. 4c), and in line with this trend, the EQE dramatically increased from 13.60% to 20.83% (Fig. 4d). The maximum luminance of this device is about 6.0 × 10^4^ cd m^−2^, which is relatively high comparing with that in the previous study (Table S4). The target PeLEDs exhibit remarkable reproducibility, as evidenced by an average EQE of 19.84% obtained from 12 devices (Fig. 4e), with a relatively low standard deviation of 2.43%. This performance surpasses that of the control PeLEDs, which only achieve an average EQE of 12.84% and exhibit a higher relative standard deviation of 11.3%. To further investigate the behavior of charge carriers in the PeLEDs, capacitance–voltage (*C-V*) measurements were performed on the control and target devices, as shown in Fig. S9 [39, 40]. In region 1, as the applied voltage approaches 0 V, the injected charge carriers into the PeLEDs can be neglected. At this point, the capacitance *C*_*1*_ of the PeLEDs can be considered as its geometric capacitance. Moving to regions 2–4, the applied voltage increases, resulting in the injection of charge carriers into the PeLEDs. In region 2, where the applied voltage is relatively small, the presence of energy barriers between different materials (such as interface barriers) makes it difficult for injected charge carriers to overcome them. Consequently, charge carriers accumulate at the interface of the two materials, and the corresponding capacitance *C*_*2*_ of the PeLEDs can be seen as its barrier capacitance. Once the applied voltage reaches a sufficiently high level, ranging from 3.0 to 3.6 V, more and more charge carriers overcome the energy barriers, leading to the diffusion and transport of charge carriers within the PeLEDs. Therefore, the capacitance *C*_*3*_ of the PeLEDs can be attributed to their diffusion capacitance. In region 4, when the applied voltage reaches the conduction voltage of the PeLEDs (specifically 3.6 V), a small number of charge carriers are injected into the perovskite layer and recombined with the majority of charge carriers. Due to the significant recombination of electrons and holes, the charge decreases sharply, resulting in a larger slope in the capacitance. Additionally, due to the strong emission of the PeLEDs we prepared, *C*_*4*_ even becomes negative. Figure S9 shows that the diffusion capacitance (region 3) of the target PeLEDs is slightly lower than that of the control, indicating that the target devices possess lower interface barriers compared to the control device. This can be attributed to the smoother film morphology of the target films and more uniform interface contact. In region 4, the target PeLEDs exhibit a steeper slope, indicating a faster carrier recombination rate, which is also favorable for device performance.

Furthermore, the EL peak position of target PeLED is slightly redshift comparing with the control device, which is consistent with the PL spectra (Fig. 4f) [41]. It is noteworthy that under a bias voltage of 4 V, the KBr-modified target PeLED device exhibited a significant enhancement of the maximum electroluminescence luminance (EL max), achieving an approximately 2.5-fold increase compared to the control perovskite-based PeLED. Figure S10 illustrates the evolution of electroluminescence of control and target PeLEDs under bias voltages ranging from 0 to 8 V. As the bias voltages gradually increase, the EL intensity of PeLEDs is also increased, without any discernible shift in peak position, exhibiting the exceptional stability of the devices. The control and target PeLEDs both emit pure green light, with narrowband emission and exceptional color purity, as evidenced by their Commission Internationale de L'Eclairage (CIE) chromaticity coordinates of (0.204, 0.752) and (0.215, 0.749), respectively (Fig. S11). We have conducted lifetime testing of the devices at high initial brightness (L_0_) of ~ 6000 cd m^−2^ under continuous constant current drive, as shown in Fig. S12, and the half lifetime (*T*_*50*_) of target device is increased by 3-folds compared with that of control device.

## Conclusions

In summary, this work demonstrated a synergistic passivation strategy by introducing ionic compounds into the precursor solution for enhancing the optoelectronic properties of perovskite thin film emitters and the performance of green PeLEDs. Through the comprehensive quantitative analysis of the increment of radiative recombination efficiency and decrement of intrinsic trap state density in target films, we concluded passivated mechanism of these ionic additives consists of two aspects: filling bromine vacancy defects with bromide anions and screening charged defects with potassium cations. This facile approach significantly enhances the optoelectronic properties of perovskite materials, promoting the device performance from 13.60% to 20.83%. The champion device exhibits the simultaneously high-performance parameter with a maximum luminance of ~ 60,000 cd m^−2^ and maximum current efficiency of 90.98 cd A^−1^. Therefore, this study introduces a new paradigm for mitigating the detrimental effects of defects, while establishing pathways for further reducing non-radiative recombination losses in perovskite materials through a synergistic approach with amphoteric ions.

## Supplementary Information

Below is the link to the electronic supplementary material.

Supplementary file 1 (DOCX 2548 kb)
