# Supplementary material for: Enhancing the Performance of Perovskite Light-Emitting Diodes via Synergistic Effect of Defect Passivation and Dielectric Screening
Source: Nanomicro Lett. 2024 May 31;16:205. doi: 10.1007/s40820-024-01405-5 (PMC11143140; doi:10.1007/s40820-024-01405-5)
Supplement: Supplementary file 1 — (DOCX 2548 kb) [file 40820_2024_1405_MOESM1_ESM.docx]

Supporting Information for

**Enhancing the Performance of Perovskite Light-Emitting Diodes via Synergistic Effect of Defect Passivation and Dielectric Screening**

Xuanchi Yu^1, 2^, Jia Guo^1,^ *, Yulin Mao^1^, Chengwei Shan^2^, Fengshou Tian^2^, Bingheng Meng^2^, Zhaojin Wang^2^, Tianqi Zhang^1^, Aung Ko Ko KYAW^2^, Shuming Chen^2^, Xiaowei Sun^2^, Kai Wang^2^, Rui Chen^2,^ * and Guichuan Xing^1,^ *

^1^ Institute of Applied Physics and Materials Engineering, University of Macau, Avenida da Universidade, Taipa, Macao 999078, P. R. China

^2^ Department of Electrical and Electronic Engineering, Southern University of Science and Technology, Shenzhen 518055, P. R. China

* Corresponding authors. E-mail: [gcxing@um.edu.mo](mailto:gcxing@um.edu.mo) (Guichuan Xing); [chenr@sustech.edu.cn](mailto:chenr@sustech.edu.cn) (Rui Chen); [jiaguo@um.edu.mo](mailto:jiaguo@um.edu.mo) (Jia Guo)

**Supplementary Figures and Tables**


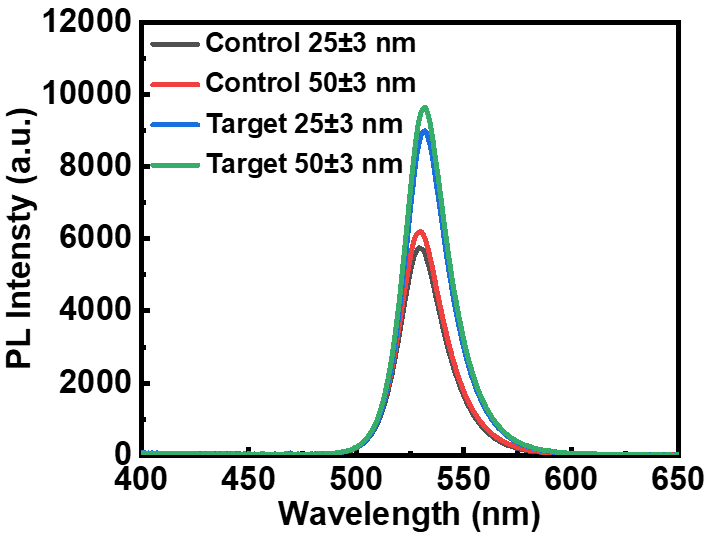


**Fig. S1** PL spectra of control and target perovskite films with different thickness


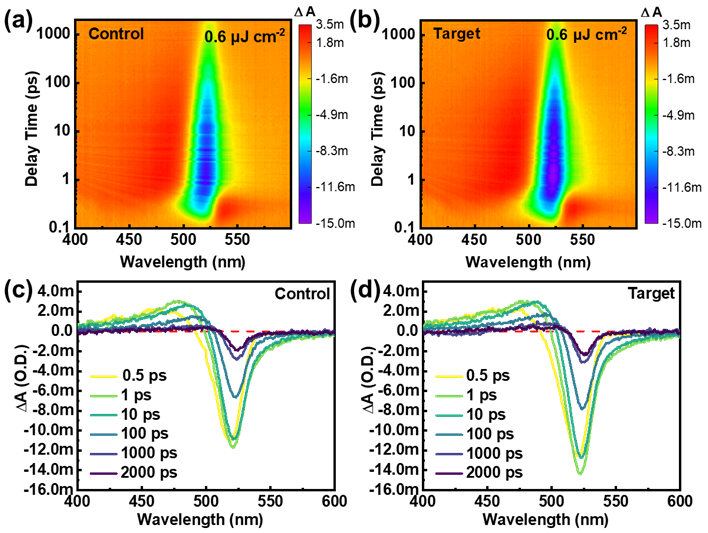


**Fig. S2** TA measurements for control and target perovskite films following excitation at 360 nm. Pseudo color TA spectra of the **a** control and **b** target films. TA spectra at selected probe delay times for the **c** control and **d** target films


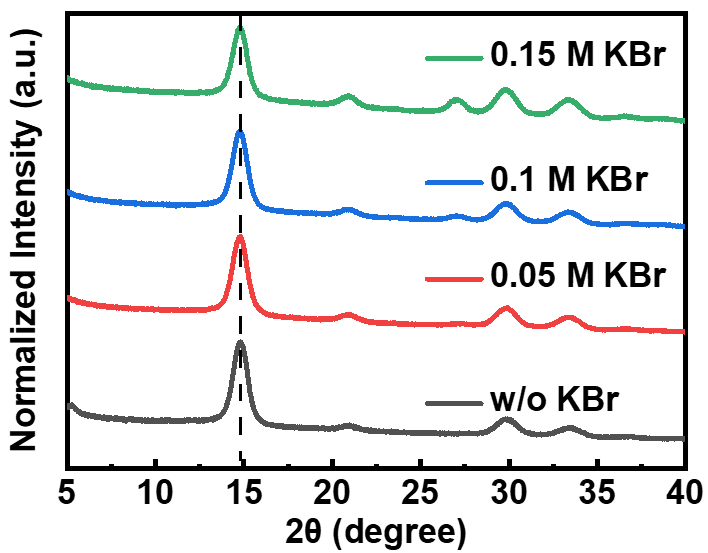


**Fig. S3** XRD patterns of quasi-2D perovskite films without and with different KBr concentration


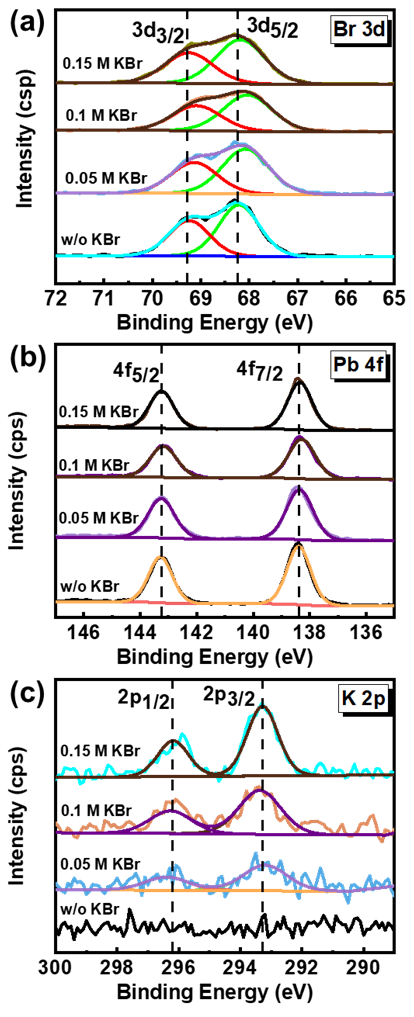


**Fig. S4** XPS spectra of **a** K 2p signals, **b** Pb 4f signals, **c** Br 3d signals and **d** N 1s signals of perovskite films without and with different KBr concentration


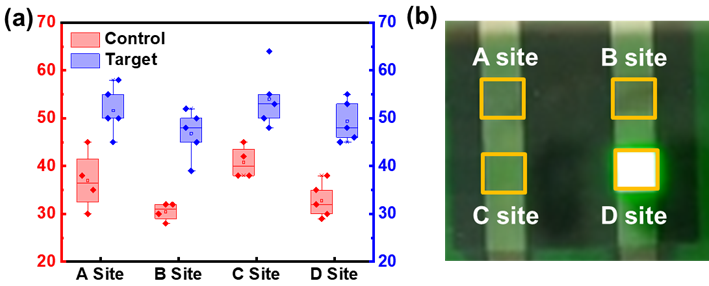


**Fig. S5 a** Average thickness of the control and target perovskite thin films at four different sites. **b** The distribution of four sites in the actual film


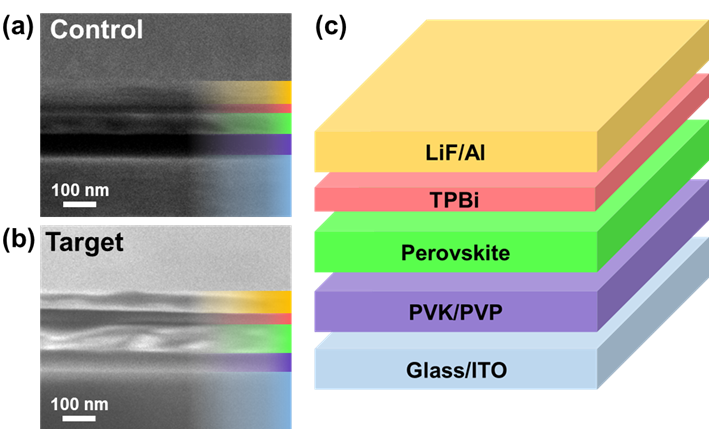


**Fig. S6** Cross-sectional SEM images of **a** control and **b** target PeLEDs. **c** Device structure of fabricated PeLEDs


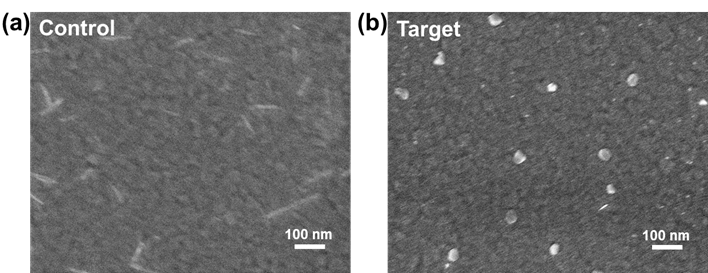


**Fig. S7** SEM images of **a** control and **b** target perovskite films


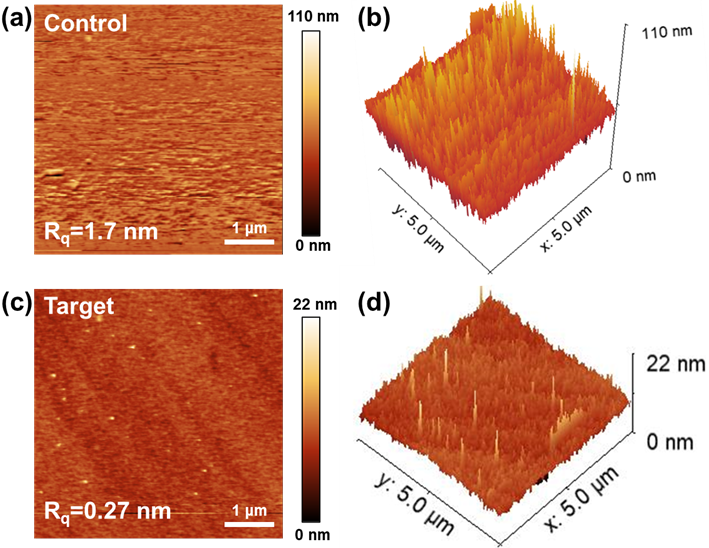


**Fig. S8** AFM **a** 2D and **b** 3D images of control perovskite films. AFM **c** 2D and **d** 3D images of target perovskite films


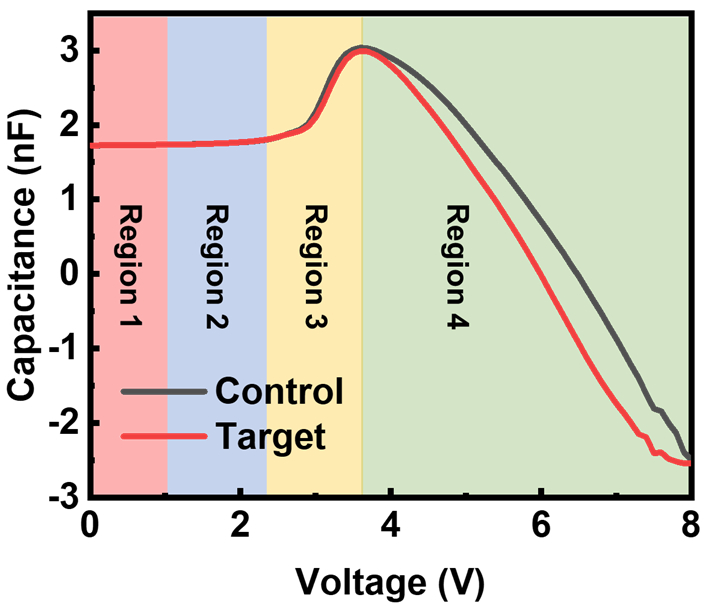


**Fig. S9** Capacitance-Voltage (*C–V*) curve of control and target PeLEDs


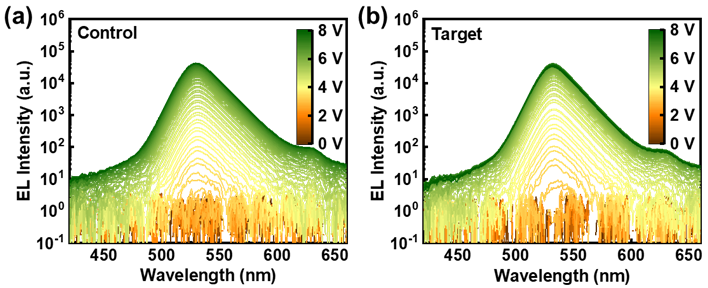


**Fig. S10** EL spectra of **a** control and **b** target PeLEDs at 0 to 8 V bias with 0.1 V interval


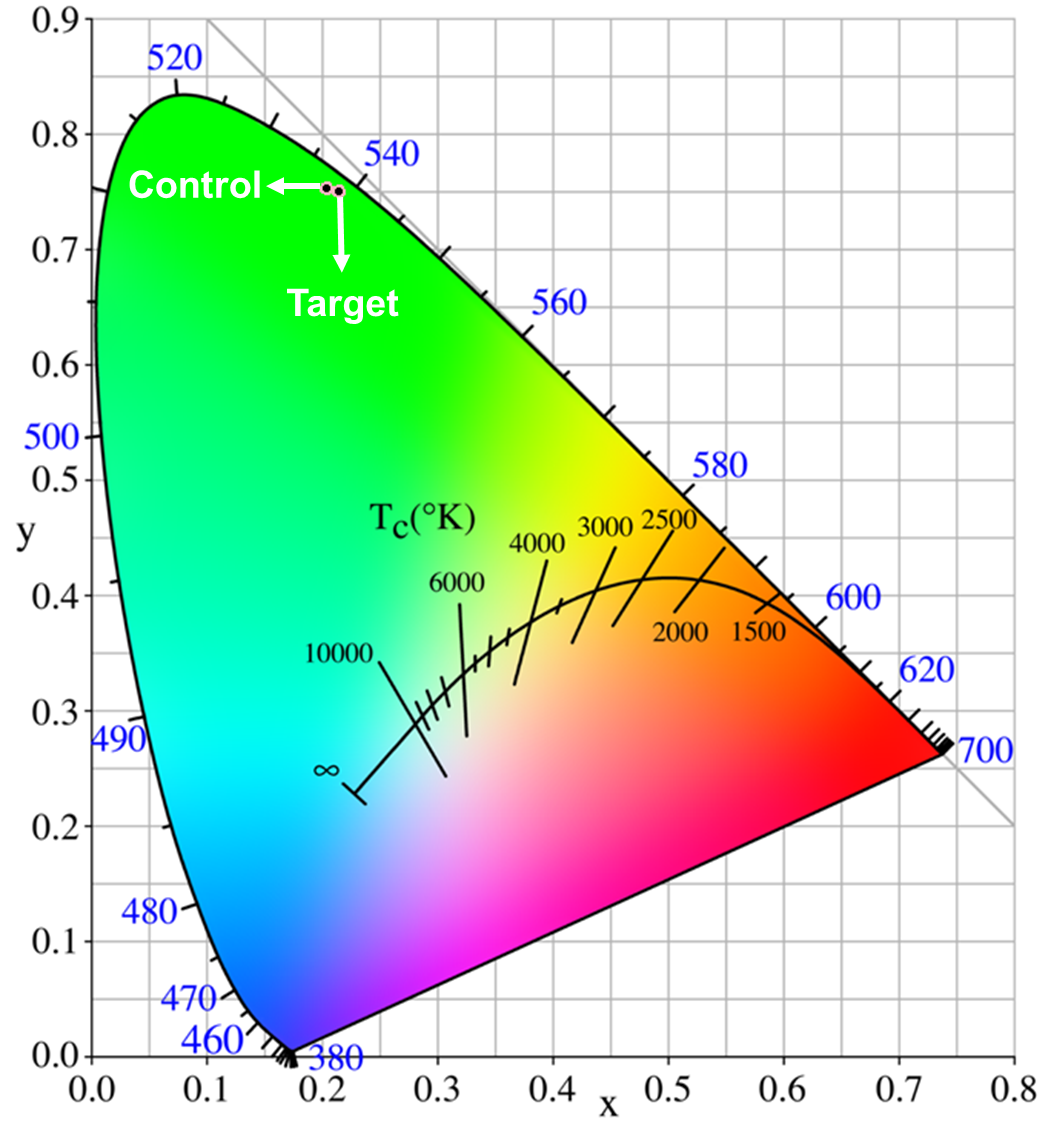


**Fig. S11** CIE coordinate of control and target PeLEDs


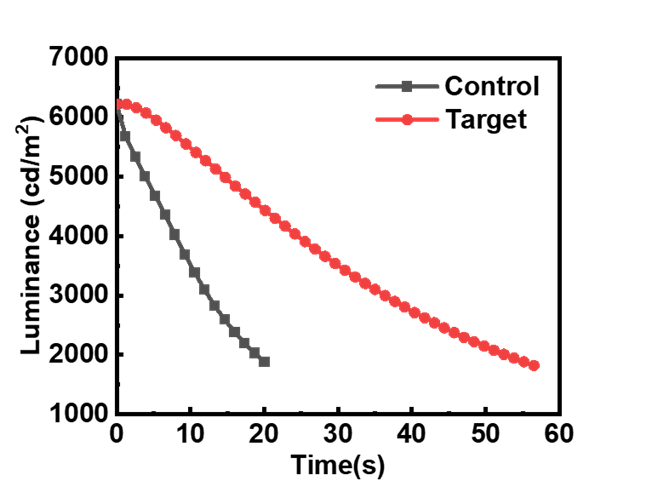


**Fig. S12** Operational lifetime tested at a constant current density of 1.6 mA cm^–2^ for control and 1.3 mA cm^–2^ for target PeLEDs

**Table S1** Lifetimes extracted from the TRPL decays of the control and target films, excited by a 360 nm laser

|  | Control | Target |
| --- | --- | --- |
| A_1_ | 0.42 ± 0.02 | 0.37 ± 0.02 |
| *τ_1_* (ns) | 9.33 ± 0.69 | 9.19 ± 0.87 |
| A_2_ | 0.56 ± 0.02 | 0.63 ± 0.02 |
| *τ_2_* (ns) | 58.95 ± 2.18 | 67.45 ± 2.63 |
| *τ_avg_* (ns) | 53.69 | 63.10 |

**Table S2** XPS fitting results of perovskite films without and with different KBr concentration

|  | C | N | Pb | Br | K | Atomic ratio (C:N:Pb:Br:K) |
| --- | --- | --- | --- | --- | --- | --- |
| w/o KBr | 34.71 | 15.65 | 9.4 | 40.24 | 0 | 3.7: 1.7: 1: 4.3: 0 |
| 0.05 M KBr | 32.44 | 14.16 | 8.99 | 40.48 | 3.92 | 3.6: 1.6: 1: 4.5: 0.4 |
| 0.1 M KBr | 38.78 | 12.02 | 7.39 | 35.17 | 6.64 | 5.2: 1.6: 1: 4.8: 0.9 |
| 0.15 M KBr | 34.31 | 11.64 | 7.58 | 39.29 | 7.18 | 4.5: 1.5: 1: 5.18: 0.95 |

**Table S3** PLQY, PL average lifetimes (*τ_avg_*), radiative decay rates (*k_r_*), and nonradiative recombination rates (*k_nr_*) of control and target perovskite films

|  | Control | Target |
| --- | --- | --- |
| PLQY (%) | 66.5 | 89.5 |
| *τ_avg_* (ns) | 53.69 | 63.10 |
| *k_r_* (🞩10^7^ s^-1^) | 1.24 | 1.42 |
| *k_nr_* (🞩10^7^ s^-1^) | 0.624 | 0.166 |

**Table S4** Performance metrics of the state-of-the-art green PeLEDs

| Perovskite | EQE  (%) | Luminance  (cd/m^2^) | CE  (cd/A) | *V*_on_  (V) | References | |
| --- | --- | --- | --- | --- | --- | --- |
| CsPbBr_3_:PEABr = 1:0.4 | 32.1 | 1.1 × 10^4^ | 111.7 | 2.5 | *Adv. Mater*. 2024, 2400421 |  |
| PEA_2_(FAPbBr_3_)_2_PbBr_4_ | 30.84 | 3.9× 10^4^ | 115.32 | ~3 | *Adv. Mater.* 2023, 35, 2302283 |  |
| PEA_2_(FAPbBr_3_)_2_PbBr_4_ | 20.82 | ~1 × 10^4^ | ~82 | ~2.8 | *Nano-Micro Lett*. 2023, 15, 119 | |
| PEA_2_FA*_n_*_-1_Pb*_n_*Br_3_*_n_*_+1_  (*n* = 5) | 21.2 | > 6.1 × 10^4^ | - | ~3 | *Adv. Funct. Mater*. 2021, 31, 2103890 | |
| PEA_2_FA*_n_*_-1_Pb*_n_*Br_3_*_n_*_+1_  (*n* = 5) | 22.2 | 5.4 × 10^4^ | - | 3 | *Light Sci Appl*. 2022, 11, 69 | |
| PEA_2_Cs*_n_*_-1_Pb*_n_*Br_3_*_n_*_+1_ | 21.0 | 4.8 × 10^4^ | - | 2.9 | *J. Phys. Chem. C*. 2023, 127, 19, 9388–9396 | |
| PEA_2_(FAPbBr_3_)_2_PbBr_4_ | 25.9 | 2.9 × 10^4^ | 108.1 | 2.9 | *Nano Lett*. 2023, 23, 23, 11082–11090 | |
| CsPbBr_3_:PEABr = 1:0.4 | 19.3 | 1.2 × 10^4^ | 61.7 | 2.6 | *Adv. Optical Mater*. 2022, 10, 2200276 | |
| **PEA_2_FA*_n_*_-1_Pb*_n_*Br_3_*_n_*_+1_**  **(*n* = 5)** | **20.83** | **5.9 × 10^4^** | **90.98** | **< 3** | **Our work** | |
